# Adjunctive S100A8/A9 Immunomodulation Hinders Ciprofloxacin Resistance in *Pseudomonas aeruginosa* in a Murine Biofilm Wound Model

**DOI:** 10.3389/fcimb.2021.652012

**Published:** 2021-04-12

**Authors:** Anne S. Laulund, Franziska Schwartz, Hannah Trøstrup, Kim Thomsen, Lars Christophersen, Henrik Calum, Oana Ciofu, Niels Høiby, Claus Moser

**Affiliations:** ^1^ Department of Clinical Microbiology, Copenhagen University Hospital, Copenhagen, Denmark; ^2^ Department of Plastic Surgery, Zealand University Hospital, Copenhagen, Denmark; ^3^ Department of Clinical Microbiology, Hvidovre Hospital, Hvidovre, Denmark; ^4^ Department of Immunology and Microbiology (ISIM), University of Copenhagen, Copenhagen, Denmark

**Keywords:** biofilm, pseudomonas, chronic wounds, resistance development, ciprofloxacin

## Abstract

**Objective:**

*Pseudomonas aeruginosa* is known to contribute to the pathogenesis of chronic wounds by biofilm-establishment with increased tolerance to host response and antibiotics. The neutrophil-factor S100A8/A9 has a promising adjuvant effect when combined with ciprofloxacin, measured by quantitative bacteriology, and increased anti- and lowered pro-inflammatory proteins. We speculated whether a S100A8/A9 supplement could prevent ciprofloxacin resistance in infected wounds.

**Method:**

Full-thickness 2.9cm^2^-necrosis was inflicted on 32 mice. On day 4, *P.aeruginosa* in seaweed alginate was injected sub-eschar to mimic a mono-pathogenic biofilm. Mice were randomized to receive ciprofloxacin and S100A8/A9 (n=14), ciprofloxacin (n=12) or saline (n=6). Half of the mice in each group were euthanized day 6 and the remaining day 10 post-infection. Mice were treated until sacrifice. Primary endpoint was the appearance of ciprofloxacin resistant *P.aeruginosa*. The study was further evaluated by genetic characterization of resistance, means of quantitative bacteriology, wound-size and cytokine-production.

**Results:**

Three mice receiving ciprofloxacin monotherapy developed resistance after 14 days. None of the mice receiving combination therapy changed resistance pattern. Sequencing of fluoroquinolone-resistance determining regions in the ciprofloxacin resistant isolates identified two high-resistant strains mutated in *gyrA* C248T (MIC>32µg/ml) and a *gyr B* mutation was found in the sample with low level resistance (MIC=3µg/ml). Bacterial densities in wounds were lower in the dual treated group compared to the placebo group on both termination days.

**Conclusion:**

This study supports the ciprofloxacin augmenting effect and indicates a protective effect in terms of hindered ciprofloxacin resistance of adjuvant S100A8/A9 in *P.aeruginosa* biofilm infected chronic wounds.

## Introduction

1-2% of our population in developed countries will acquire a chronic wound during their lifespan and up to 78% of these have been reported to be associated with bacteria in a biofilm formation (de Oliveira et al., 2017; Malone et al., 2017; Martinengo et al., 2019). Such non-healing wounds will have significant physical-, social- and psychic consequences for the individual patient, in addition to the burden on the worldwide healthcare budget (Gueltzow et al., 2018).

Biofilm infections with Pseudomonas aeruginosa, one of the ESCAPE pathogens (*Enterococcus faecium*,* Staphylococcus aureus*,* Klebsiella pneumoniae*,* Acinetobacter baumannii*,* P. aeruginosa*, and* Enterobacter* species) are associated with chronic wounds (Rice, 2008), affect the immune homeostasis and suppress neutrophil markers (Trøstrup et al., 2017b).

When polymorphonuclear leukocytes (PMN) respond to the bacteria congregated in biofilms, they release reactive oxygen species and matrix metalloproteases (MMP) that incompletely degrade the biofilm matrix. When the biofilm persists and the PMN attraction is prolonged, an overstimulation of these oxygen species and proteinases cause collateral damage to surrounding healthy tissue. MMPs are balanced by tissue inhibitors of metalloproteinases (TIMPs), but in chronic wounds this balance is disrupted with increased levels of MMPs and reduced levels of TIMPs, which further reduces levels of wound repair growth factor. A vicious cycle continues as the rhamnolipids (i.e. rhamnolipid B) lyse PMNs, leaving the biofilm *in situ*, resulting in further chemotaxis of PMNs with continued release of proteinases. Overall, these pathological processes will result in a prolonged inflammatory phase of the wound with a defect neutrophil function, local suppression of keratinocyte chemoattractant (or keratinocytes-derived chemokine, KC), granulocyte colony-stimulating factor (G-CSF) and S100A8/A9 (also known as myeloid related protein, MRP 8/14 or calprotectin) (Trøstrup et al., 2017a) as well as providing the biofilm with nutrients from the lysed immune cells.

With the aim of synergistic activity or decreased risk of resistance development, dual antibiotics, often a beta-lactam combined with an aminoglycoside, polymyxin or fluoroquinolone, are considered our best option in the most recalcitrant cases involving *P. aeruginosa*.

The immunosuppressants S100A8 and S100A9 are part of the alarmin- or damage-associated molecular patterns (DAMP) – group. They can bind calcium reversibly and thus facilitate regulation of different cellular functions. Functionally, the S100A8/A9 complex is released into the microenvironment from the cytosol of neutrophils and monocytes when the wound (and later infection) is established. This multifaceted protein changes from having a physiological homeostatic function within the cell to sensing the extracellular environment and triggering pattern recognition receptors (Toll like receptor 4) on innate cells with a succeeding conformational change followed by pro-inflammatory cytokine release (Wang et al., 2018). In addition, S100 proteins are also known to play a role in the cytosol tubulin polymerization and cytoskeleton rearrangement (Pirr et al., 2017), and to have a substantial antimicrobial activity towards different microbes by binding of manganese, iron and zink (Wang et al., 2018).

Resistance against the fluoroquinolone ciprofloxacin has been reported to increase up to 5-fold when zinc is present and that a zinc supplementation *in vitro* attenuates the antimicrobial activity of S100A8/A9. Manganese is an essential nutrient and plays a role for different microbial enzymes involved in metabolism and oxidative stress and is a known catalyst for ciprofloxacin degradation. Lastly, iron is an essential nutrient for all microbial pathogens. Overall it is known that S100A8/A9 has antimicrobial activity against microbes with different manganese and iron requirements (Wang et al., 2018).

Our group has previously demonstrated that S100A8/A9 is a promising antimicrobial adjuvant when combined with ciprofloxacin in a murine chronic infection/wound model, measured by increased bacterial killing, PMN related cytokines, increased anti-inflammatory, and lowered pro-inflammatory proteins after three days of treatment. This detected adjuvant effect of S100A8/A9 has not been observed *in vitro* indicating that the effect is dependent on host cells (Laulund et al., 2019).

With an increasing number of *P. aeruginosa* isolates becoming Carbapenem-resistant, and the established adjunctive wound treatment including hyperbaric oxygen therapy (HBOT) still considered “high effort”, easy and low-cost adjunct treatments are necessary. As stated above, we showed S100A8/A9 can serve as an adjunctive compound to ciprofloxacin in *P. aeruginosa* in the early phases of biofilm infected chronic wounds. In the present setup, we speculated whether this combination therapy provided for a longer period, inhibits ciprofloxacin resistance development and arrests the ongoing pathological pro-inflammatory response in an established murine wound model and thus improves healing.

## Materials and Methods

### Ethics

The experiment is approved by the Animal Ethics Committee of Denmark (approval number 2015-15-0201-00618) and all animal handling was at all times performed in accordance to the National, European and ARRIVE guidelines by trained professionals.

Planning of this study was based on guidelines from the ethical animal committee. The animals could not be replaced by *in vitro* analysis because of the need for host response analyses.

### *In Vivo* Study Design

**Figure 1:**
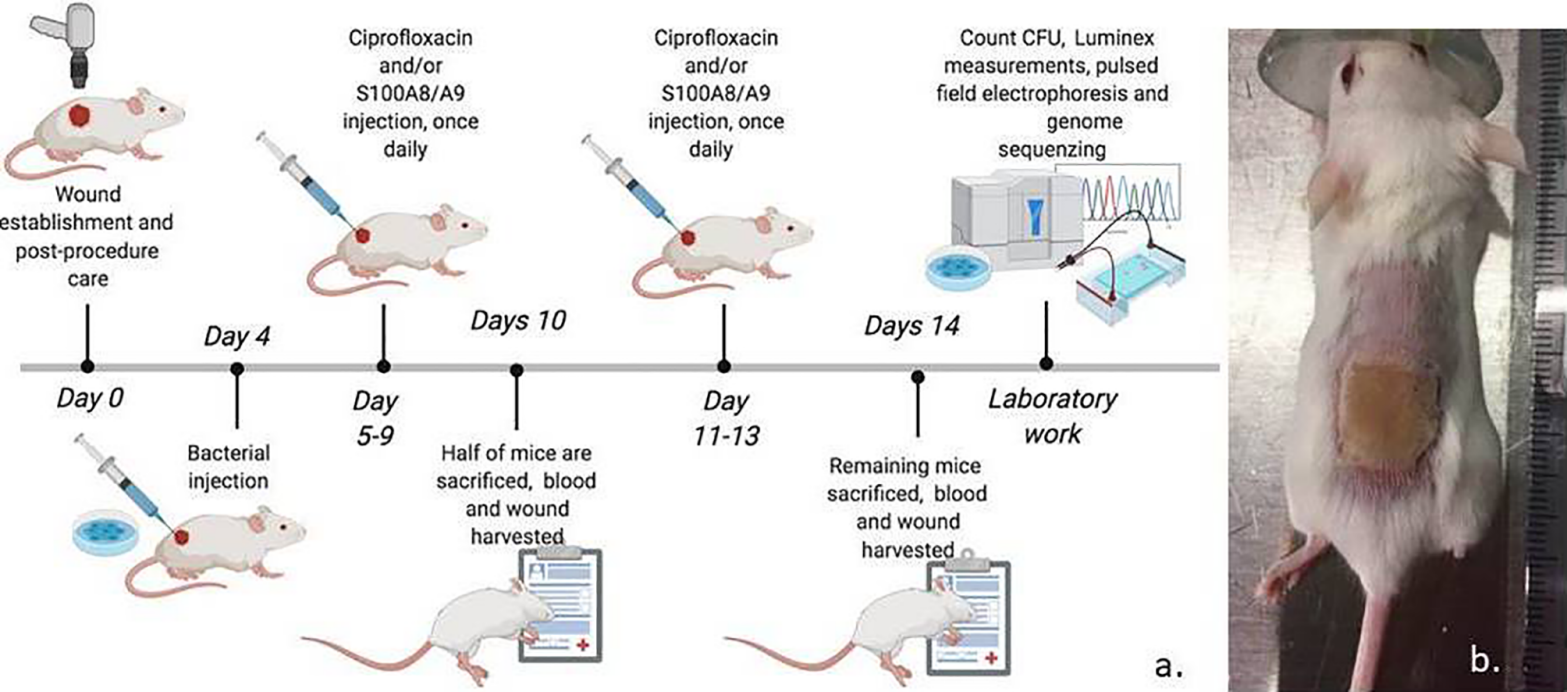
**(A)** Experimental setup. 32 BALB/C 12-week-old mice were applied a full thickness necrosis by burning under analgesia/anesthesia and infected with pseudomonas aeruginosa embedded in alginate to mimic a biofilm. The mice were randomized into three treatment groups: 1) Ciprofloxacin and S100A8/A9 (n=14). 2) Ciprofloxacin monotherapy and placebo saline (n=12). 3) Saline controls (n=6). Half of the mice in the three groups were terminated day 10 post-burn (day 6 of infection) and the remaining on day 14 (day 10 of infection). All mice were treated until their sacrifice. **(B)** Wounds were photographed during and after sacrifice for wound size evaluation. Wounds were collected and homogenized for further quantitative bacteriology, host factor measurements by means of Luminex and resistance testing. Bacteria from three mice wounds developed Pseudomonas resistance by Etest. These were further processed with pulsed field electrophoresis and whole genome sequencing (Created with BioRender.com).

Thirty-two BALB/C pathogen-free 12-week-old female mice (Janvier, CS 4105 Le Genest Saint Isle, Saint Berthevin, Cedex 53941 France) were acclimatized in the laboratory (Biotech Research & Innovation Centre, University of Copenhagen, Denmark) one week prior to the experiment start (**Figure 1**). The sacral area of each mouse was shaved and a full thickness burn (2.9 cm2) was inflicted with a reproducible and standardized method by hot air (Easyheat 500, Robert Bosch GmbH, Germany) for 5 seconds after administering 0.1 mL/10 g s.c, Hypnorm/Midazolam (mixture of 1 mL Fentanylcitrate 0.315 mg/mL + Fluanisone 10 mg/mL, 2 mL sterile water and 1 mL Midazolam 5mg/mL, Hameln Pharma ltd.) per mouse (Calum et al., 2014). After the burn-procedure, 200 µl NaCl 0.9% was injected in the neck region to prevent dehydration and mice were placed in individual ventilated cage on heat mats adjusted to 35°C to avoid hypothermia. Location of the burn did not hinder the natural behavior of the animals. To prevent potential dolor from the area bordering up to the burn, analgesia with repeated subcutaneous (s.c.) injections of buprenorphine (0,1 mg/kg) was administered 24 hours post burn procedure. The bordering area was previously examined histopathologically to be less than <100 µm (Calum et al., 2017).

*P. aeruginosa* produces the exopolysaccharide alginate when settling in a biofilm in nature. The bacterial biofilm growth was mimicked by preforming the matrix by means of seaweed alginate around the bacteria (Christophersen et al., 2012). If we inject the bacteria in the planktonic mode of growth, they will disseminate and result in sepsis in a number of mice, before they have developed a local biofilm. This embedment was performed by centrifuging and resuspending an overnight PAO1 culture, and further mixing it into a 1% alginate solution. This solution was pumped out from a syringe through an encapsulation nozzle generating a vapor of bacterial containing seaweed alginate beads, capured in a 0.1M TRIS HCl buffer containing 0.1 M CaCl_2_ (Pump: model 3100; Graseby, United Kingdom. Nozzle: Var J30; Nisco Engineering, Zurich, Switzerland). After a bead washing procedure, the CFU were determined by dissolving the beads in citrate buffer followed by plating. Then, beads were diluted to reach a bacterial concentration of 1x10^7^ ready for wound injection. Thus, on the fourth day post thermal damage, 100 µL of a 10^7^ colony forming units (CFU)/mL *P. aeruginosa* (strain PAO1, Iglewski strain) solution was injected under the eschar to produce a mono-pathogenic biofilm infection.

The 32 mice were randomized into three groups:

1. Ciprofloxacin and S100A8/A9 (n=14)
2. Ciprofloxacin monotherapy and placebo saline (n=12)
3. Saline controls (n=6).

The dosage of ciprofloxacin was 500 µL (2 mg/ml) (Fresenius Kabi, Islands Brygge 57, 2300 KBH S, Denmark) injected s.c. on the abdomen. This dosage has previously been determined to be sub-optimal as monotherapy for killing of biofilm growing *P. aeruginosa* in the chronic wounds and resembles the clinical observation of poor efficacy of ciprofloxacin monotherapy against biofilm infections. Ciprofloxacin represents, along with a few other fluoroquinolones, the only anti-*pseudomonas* antibiotics administered orally and chosen because of this particular administration form. The mice received the antibiotics systemically to ensure the same dosing in all animals, but also to avoid the traditional method of oral gavage that is assumed stressful, and can induce significant oesophageal damage in the animal.

Mice receiving recombinant murine S100A8/A9 (Cloud Clone, Katy, TX, USA) were administered 200 µL (5 µg/mL) given as sub eschar injections. Half of the mice in the three different groups were terminated day 10 post-burn (day 6 of infection) and the remaining on day 14 (day 10 of infection). All mice were treated until their sacrifice. The general condition of the mice was evaluated by a daily clinical scoring (Langford et al., 2010).

### Digital Photoplanimetry

Healing was estimated by planimetry with photos of wounds on day 5, 10 and 14 post-burn-procedure. In order to standardize this procedure with no potential skin traction, mice were temporarily sedated in an isoflurane inhalation box or photographed postmortem. Digital image analysis was performed by two independent researchers using Image J (Bethesda, MD, USA).

### Bacterial Burden and Resistance Development

All wounds were removed in toto postmortem using a standardized technique, ensuring all wounds were comparable, and then individually homogenized in 1,0 mL saline for 20 seconds at 14.000 rpm (Heidolph Silent Crusher M, Heidolph Instruments, Schwabach, Germany (Blender, company), serial diluted and underwent quantitative bacteriology evaluation by plating. CFU were counted manually by two independent researchers and the result was adjusted for the dilution factor in each homogenate. E-tests (Biomérieux, Ballerup, Denmark) were performed on each wound homogenate. Isolates from the three mice that developed ciprofloxacin resistance (mice no.21, 25 and 26) were sent to pulsed field gel electrophoresis and sequencing at the Department of Immunology and Microbiology (ISIM) at the University of Copenhagen. This was performed to clarify the causal genetic mechanisms behind the developed antimicrobial resistance.

Cytokines and growth factors in the supernatant of the homogenized wound tissue were measured by LUMINEX^®^ 200TM Platform (Luminex Corp., Austin, TX, USA). Customized bead-based multiplex assays (Biotechne R&D Systems, 614 McKinley Place NE, Minneapolis, MN 55413) were used to quantify components of the wound homogenate. We included measurement of Tumor necrosis factor (TNF)-α, Tumor necrosis factor-like weak inducer of apoptosis (TWEAK), Interleukin (IL-1β). Chemokine/keratinocyte chemoattractant (CXCL1/KC), Osteopontin (OPN), S100 calcium-binding proteins (S100A8 and S100A9), Granulocyte-colony stimulating factor (G-CSF) and tissue inhibitor of metalloproteinases-1 (TIMP-1).

### Sequencing of Fluoroquinolone Resistance Determining Regions

DNA of the ciprofloxacin resistant isolates was purified and *gyrA*, *gyr* B and the negative regulator of MexCDoprD efflux pump *nfx*B were amplified and sequenced as previously described (Jørgensen et al., 2013).

### Statistics

Non-parametric statistical methods were employed; including the Kruskal-Wallis by ranks for multiple comparisons and Mann-Whitney tests were used for dichotomously comparing groups. P ≤ 0.05 was considered statistically significant.

Statistical analyses on the cytokines were performed directly on the fluorescence index (mean of duplicates) after subtracting the fluorescence background level on all samples, to avoid any faultily computerized estimated values. When measurements were outside the standard curve, repeated Luminex measurements were performed on different sample dilutions (this was performed on S100A8 and TIMP-1). Bacterial wound densities (Medians and 95% confidence intervals) were calculated as CFU/wound.

Statistics were performed using Microsoft Excel (version 15.41, WA, USA) and GraphPad Prism (version 8.0, CA, USA).

## Results

### Resistance Development

**Figure 2:**
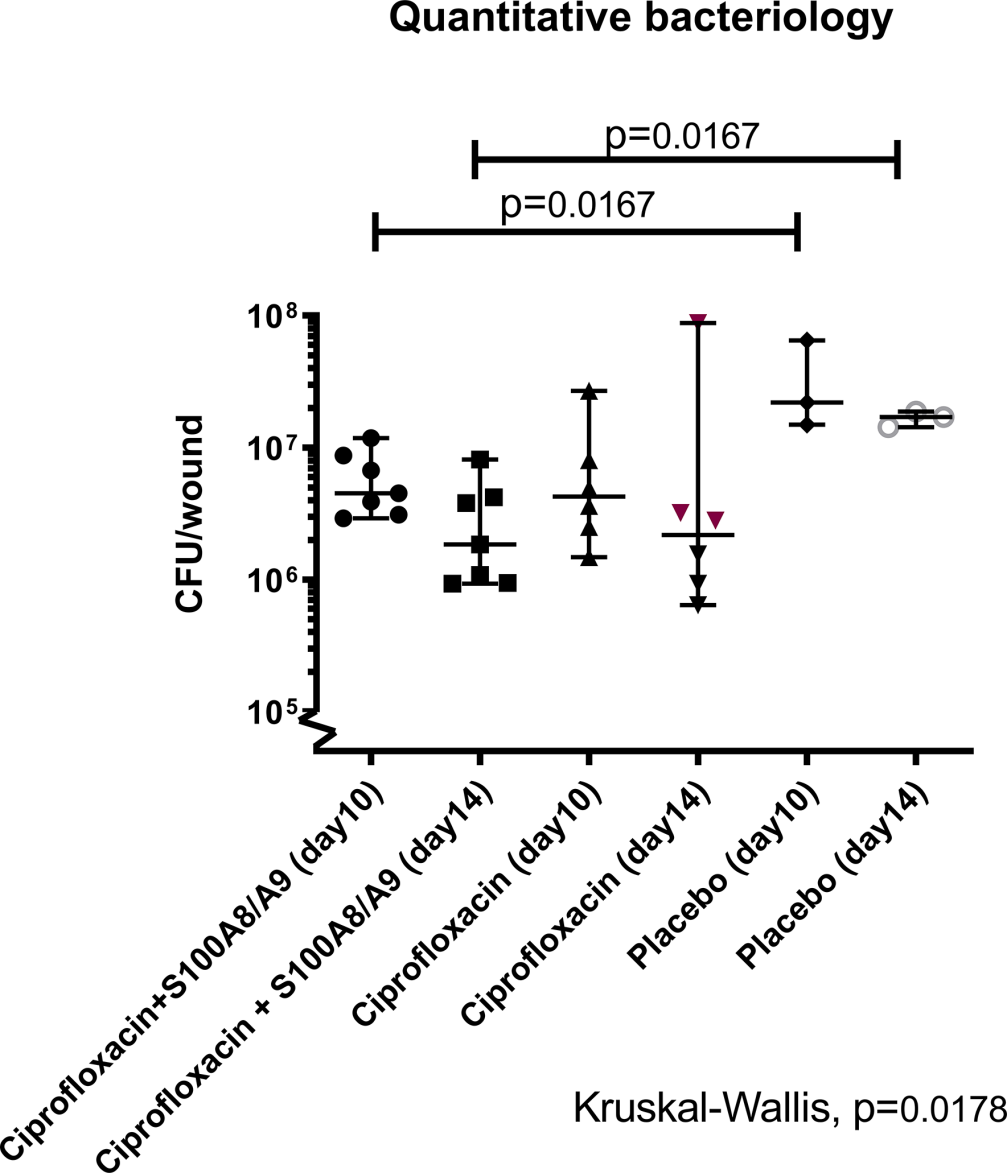
Quantification of colony forming units (CFU). 32 female Balb/C mice were inflicted with a burn-wound and 4 days post-infliction sub-escharly infected with P. aeruginosa. Treatment with S100A8/A9 (sub-eschar), ciprofloxacin (s.c.) or both was applied once daily. Half of the animals from each group was sacrificed on day 10 post-burn (day 6 after infection) and the other half on day 14 post-burn (day 10 after infection). Wounds were removed in toto, homogenized and quantitative bacteriology was determined. The lowest bacterial count was detected in the dual treated group on day 14 [median 95%CI: 1.84·10^6^ (9.30·10^5^; 8.10·10^6^)] and was statistically different from the level in the placebo group [1.70·10^7^(2.43·10^6^; 1.87·10^7^)]. The same difference was detected in mice sacrificed on day 10.

**Figure 3:**
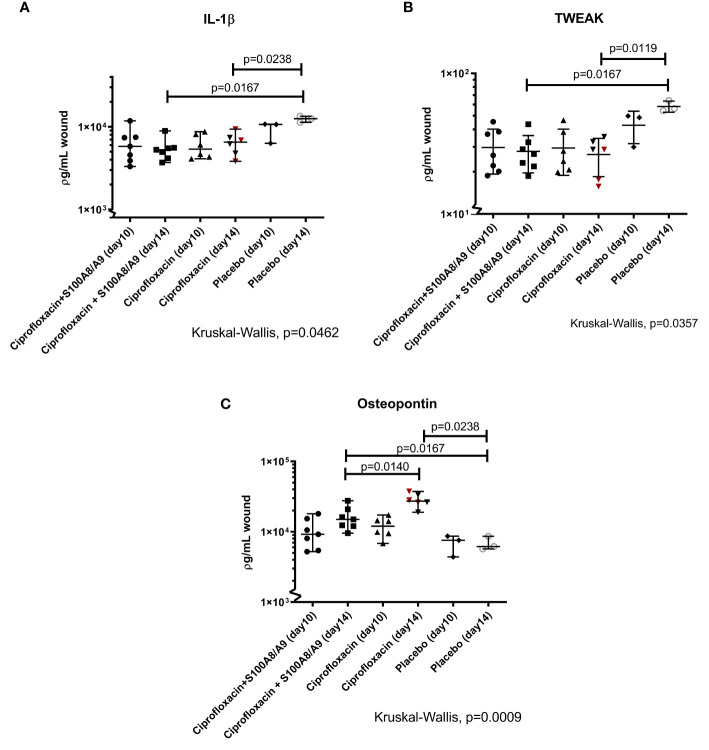
**(A–C)** IL-1β, TWEAK, and osteopontin. The wounds were harvested, homogenized and the supernatants were analyzed using a LUMINEX. Displayed are the individual measured cytokine concentrations in ρg/mL wound homogenate for every mouse) and median 95%CI. Statistically significant P values are displayed on the figure. **(A)** The pro-inflammatory IL-1β responses in the treated groups were dampened after 14 days (dual therapy: median 95%CI: 5.27·10^3^ (3.70·10^3^; 8.92·10^3^). Monotherapy: 6.53·10^3^ (3.82·10^3^; 9.56·10^3^). Placebo: 1.25·10^4^ (1.13·10^4^; 1.34·10^4^). **(B)** The TWEAK level was only statistically different from the placebo group on day 14 (dual therapy: 26.69 (18.65;43.51), monotherapy: 28.75(15.64 ;35.48) and placebo 56.13(54.26;64.18). **(C)** The level of OPN in the three groups was compared on both sacrifice-dates. Osteopontin was significantly higher in the ciprofloxacin monotherapy group on day 14 compared to the dual-treated group on day 14 [2.78·10^4^(1.90·10^4^;3.75·10^4^) and 1.50·10^4^(9.55·10^3^;2.76·10^4^)]. Both treatment groups had higher levels of OPN compared to the placebo group [6.18·10^3^(5.69·10^3^;8.62·10^4^)] on day 14.

**Figure 4:**
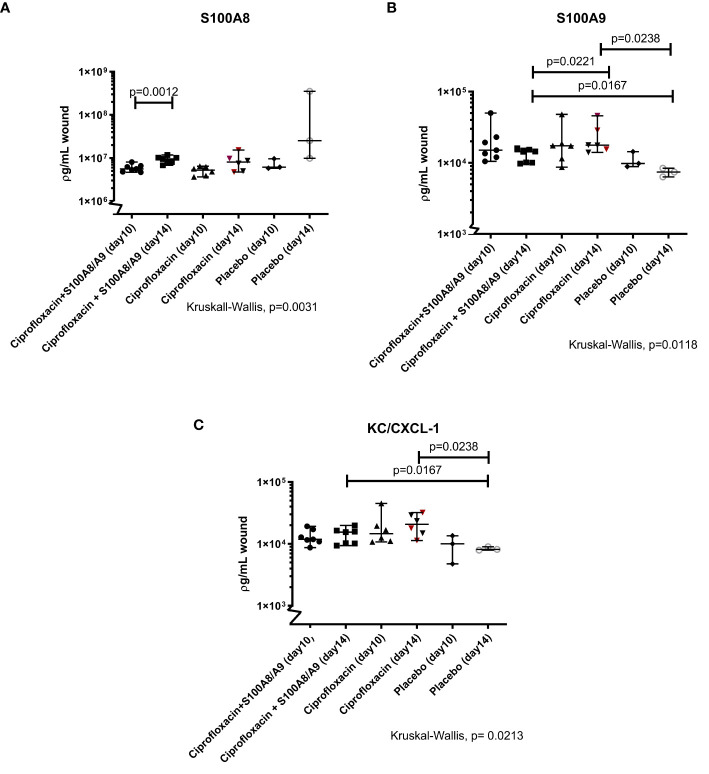
**(A–C)** Immunomodulating S100A8 and S100A9and KC/CXCL-1. Illustrated are the Luminex measurements (median 95%CI) of mice inflicted with pseudomonas infected skin necrosis randomized to sacrifice on day 10 or 14 and to different treatment regimes. Statistically significant P values are displayed on the figure. **(A)** The level of S100A8 increased from day 10 to day 14 in the dual treated group [respectively 5.57·10^6^(4.66·10^6^; 8.02·10^6^) and 9.51·10^6^ (6.76·10^6^;1.18·10^7^)]. **(B)** The concentration of the alarmin S100A9 was higher in the monotherapy group (1.77·10^4^ [1.40·10^4^; 4.58·10^4^)] compared to the dual treated group (1.43·10^3^(9.75·10^2^; 1.60·10^4^) and both treatment groups, on day 14, differed from the level in the placebo mice. **(C)** Both treatment groups had higher levels of KC/CXCL-1 on day 14, compared to the group receiving only saline [dual treated group 1.55·10^4^(9.35·10^3^;1.98·10^4^)and monotherapy group 2.07·10^4^(1.13·10^4^; 3.21·10^4^)].

In three mice receiving ciprofloxacin monotherapy the alginate-embedded *P. aeruginosa* developed resistance over the course of the 10 days of treatment. The three mice afflicted are marked red in **Figures 2**–**4**. No ciprofloxacin resistance was observed after 6 days of treatment in any groups. None of the mice receiving combination therapy or placebo changed resistance pattern. This was detected by standard E-tests. The three ciprofloxacin-resistant *P. aeruginosa* isolates identified one low-resistant strain (MIC of 3µg/mL) and two highly resistant strains (MIC >32 µg/mL, the original PAO1 strain had a MIC of 1.00 µg/mL).

The identified three resistant *P. aeruginosa* and the original strain (strain PAO1, Iglewski) were analyzed by pulsed field gel electrophoresis. Results conclude that all colonies were isotypic representing the same bacterial isolate, PAO1.

Sequencing of the three ciprofloxacin-resistant *P. aeruginosa* isolates identified a C248T mutation in the DNA gyrase *gyrA* (encoding a T83I substitution) for the two highly resistant strains and C67G and A69T causing a K23Q substitution in *gyrB* was detected in the low ciprofloxacin resistant strain.

### Quantitative Bacteriology of *P. aeruginosa* Infected Wounds

The settlement of the biofilm was successful in all three groups (*data not shown*). The multivariate analysis of the bacterial densities in the wound showed a significant difference between groups (p<0.02, **Figure 2**).

When groups were analyzed dichotomously, no difference in bacterial load was detected between the mono- and the dual-treatment groups, but the dual treated group had significantly lower bacterial wound load than the placebo group on both termination days (p=0.02 and p=0.02). In contrast, between the placebo group and ciprofloxacin monotherapy group no difference was detected on either day.

### Host Response

The levels of IL-1β (**Figure 3A**) and TWEAK (**Figure 3B**), both secreted by innate immune cells, were lower in the treated mice (IL-1β: dual treated, p=0.02 and ciprofloxacin-mono treated, p=0.02 TWEAK: dual treated, p=0.01 and ciprofloxacin-mono treated) compared to the placebo-group on day 14 of sacrifice. The level of TNF-α did not differ between groups (data not shown).

OPN was significantly higher in the ciprofloxacin monotherapy group on day 14 compared to the dual-treated group on day 14 (p=0.01) Both, the mono- and the dual treated group, had higher levels of OPN compared to the placebo group on day 14 (respectively p=0.02 and p=0.02).

The level of S100A8 significantly increased over the course of the experiment in the dual treated group (p=0.001). The same tendency has been detected in the ciprofloxacin monotherapy treated group, even though not statistically significant (p=0.09). No difference was detected between the treatment groups over the course of the experiment (day 10, p=0.5 and day 14, p=0.5) (**Figure 4A**).

Both the dual and the mono treated group had overall higher levels of S100A9 compared to the placebo group on day 14 (respectively p=0.02 and p=0.02). Interestingly, the ciprofloxacin monotherapy group displayed significantly higher levels of S100A9 on day 14 compared to the dual treated group (0.02) (**Figure 4B**).

The neutrophil chemoattractant KC/CXCL1 was also investigated. Here, both treatment groups had higher measurements on day 14, compared to the group receiving only saline (dual treatment, p=0.02, and mono-treatment, p= 0.02) (**Figure 4C**).

The measurements of TIMP-1 and G-CSF were not significantly different in any of the groups (data not shown).

### Planimetry

Digital planimetry analysis did not show any macroscopic healing differences when looking at the wound size between the groups, neither in a multi-comparison, nor in dichotomous analyses. Interestingly, the two biggest wounds belonged to mice with ciprofloxacin resistance (data not shown).

## Discussion

The management of a chronic wound is complex with the dynamic involvement of the host immune response towards pathogens, microbiota and comorbidities. Furthermore, the quality of the preexisting skin and local and/or systemic bioavailability problems caused by the physical properties of the wound have to be taken into consideration. However, the intrinsic and/or acquired antibiotic resistance mechanisms of the pathologically invading microorganism(s) have the potential to make the treatment of choice, antibiotics, obsolete. Resistance development in biofilms due to increased mutation rates and prolonged selection pressure caused by ineffective antibiotic therapies is frequent (Ahmed et al., 2018).

With our established murine model, we tested whether supplementing systemic ciprofloxacin treatment against biofilm growing *P. aeruginosa* with topical S100A8/A9 could prevent the development of antibiotic resistance in a murine model.

The group of mice receiving ciprofloxacin and the immunosuppressant S100A8/A9 did not change resistance patterns and was different from the placebo group in quantitative bacteriology on both sacrifice dates (**Figure 2**). This was in contrast to the group only receiving ciprofloxacin where *P. aeruginosa* isolates from half of the mice developed phenotypical resistance against ciprofloxacin after the longer treatment period. In addition, reduction in the pathogenic bacterial burden essential for healing was detected on both day 10 and day 14 in the dual-treated group compared to placebo. No such differences were seen in the ciprofloxacin monotherapy group. This indicates at least an additive effect of the dual treatment in terms of hindered resistance and lowered bacterial burden. In our previous work on S100A8/A9 and ciprofloxacin the differences between quantitative bacteriology in the groups were more distinct after three days of treatment, indicating a possible tolerance effect in the wound after the longer treatment periods with S100A8/A9.

The significance of findings was further supported, since the three mice that developed ciprofloxacin resistance during the course of the trial (sacrificed on day 14) had the highest bacterial burden in their wounds on the day of sacrifice (**Figure 3A**) and presented with the macroscopically biggest wounds (data not shown).

In order to evaluate the influence of adjunctive S100 therapy on the host immune response, we measured the levels of several wound related cytokines.

The first cytokines to be released upon injury are TNF-α and IL-1β with a peak within hours of insult (Kondo and Ohshima, 1996). These cytokines are considered some of the first mediators of the innate immune response, by activating the expression of adhesion molecules on endothelial cells and initiating neutrophil recruitment. IL-1β further stimulates the gene transcription and release of OPN (Mazzali et al., 2002). OPN is involved in the physiological immune response at multiple different levels, but has also been shown to mediate pathological processes in cancer, diabetes, cardiovascular diseases and in infectious diseases such as herpes simplex and listeria infection (Rittling and Singh, 2015; Icer and Gezmen-Karadag, 2018). For our study, OPN is a relevant candidate due to its chemo-attractive abilities for macrophages and T-cells. Inflammation-triggered OPN release has earlier been shown to hinder the rate of repair and contribute to wound fibrosis (Mori et al., 2008; Chimento et al., 2017). OPN stimulates a rise in Transforming Growth Factor (TGF)-α, a secretion of metalloproteinases, and fibroblast proliferation. OPN also activates the migration of mesenchymal stem cells to wounds and has been shown to have neovascularization potential (Mazzali et al., 2002; Wang et al., 2017; Icer and Gezmen-Karadag, 2018). It is mainly considered a proinflammatory protein, but also has substantial anti-inflammatory abilities.

Both treatment groups had lower levels of the proinflammatory IL-1β (**Figure 3A**) and increased OPN levels (**Figure 3C**) on day 14 (day 10 of treatments) compared to the placebo group. We believe this illustrates an increased anti-inflammatory and angiogenesis status and thus a progression from the proinflammatory phase to the proliferative phase of wound healing.

TWEAK/Fn14 increases the expression or secretion of different factors in the immune response, including IL-1β and TNF-α among others. A constant activation of the TWEAK/Fn14 pathway has been suggested to promote a dysfunctional healing response (Burkly, 2014). TWEAK has previously been shown to contribute to the inflammatory response, fibrosis, angiogenesis and tissue remodeling (Berzal et al., 2015; Liu et al., 2017; Liu et al., 2019). We found a lower level in the treatment groups on day 14 compared to the placebo group (**Figure 3B**).

The alarmins, S100A8, S100A9 and chemokine CXCL1/KC are all potent neutrophil recruiting molecules. KC is the murine homolog to the human CXCL1/IL-8 and besides being an important chemoattractant for PMNs it is also a significant angiogenesis promoter. It has been reported that one day after the insult, neutrophils constitute approximately 50% of cells in the wound bed (Gillitzer and Goebeler, 2001). As a positive reinforced loop, recruited neutrophils are responsible for increased production of KC. Although not statistically significant, our results did show a tendency for increased KC in the ciprofloxacin group.

Mice receiving supplementing S100A8/A9 did not have increased levels of S100A8 and S100A9 at study termination. This may be surprising, however it has been established that the secretion of S100A8/A9 is restricted by a negative feedback regulatory mechanism. From our previous work we estimate that this negative regulation of S100A8/A9 is functional after approximately 72 hours and therefore reveals similar levels of S100A8/A9 as measured for the ciprofloxcin monotherapy group at day 6 and day 10 of treatment. Whether increased levels of S100A8/A9 can be obtained by higher topical application remains to be investigated – but may also results in unintended sideeffects.

Although, quantitative bacteriology was most pronounced in the mice developing ciprofloxacin resistance, no general relation was observed between antibiotic resistance and the cytokines. However, the wound of one mouse in which resistance developed, had higher levels of the early phase cytokines IL-1β and TNF-α in addition to higher levels of the alarmins, S100A8 and S100A9. TIMP-1 was a factor 10 lower than the remaining group. The lack of a clear relation in mice where resistant isolates were observed may also be due to the presumed early phase of resistant development, as well as it probably did not occur simultaneously in the three mice. If investigated at a later time point with an ongoing selective pressure of ciprofloxacin therapy, the lack of infectious control could possibly have precipitated more and with a clearer relationship to the host response.

### Limits of the Study

Most chronic wounds are polymicrobially infected. This study was based on a mono-pathogenic P. aeruginosa biofilm setup in order to clarify the possible protective effect of ciprofloxacin combined with S100A8/A9. It would be interesting to investigate the two compounds in a polymicrobial model combined of nonpathogenic and pathogenic bacteria, when the effect of S100A8/A9 and ciprofloxacin on resistance development in the relevant separate species are better understood.

Our animal model is established in mice, with a different healing physiology compared to humans. These interspecies differences include acknowledged differences in immune response including a different distribution of peripheral blood leukocytes and defensins. Other differences include the number of hair follicles, presence or absence of stem cells, dermis- and epidermis thickness with adhesion to the underlying tissue in humans, but not in mice. This influences the healing method, with murine contraction healing aided by the panniculus carnosus with a weaker barrier function and enhanced percutaneous absorption versus the humane granulation healing (Zomer and Trentin, 2018). Despite these differences, mice models are considered invaluable in understanding normal and pathological healing in humans. The primary endpoint, however, showed that adding S100A8/A9 could prevent development of antibiotic resistance and this will presumably also be the case in humans. Another limitation of our study is the lack of revealing the mechanism behind the beneficial effect of supplemental S100A8/A9 in preventing development of antibiotic resistance. Although, a tolerance towards S100A8/A9 was indicated, compared to the clearer effect observed after three days of treatment, the reduced bacterial load compared to the placebo group and the longer time for development of resistance does indicate an additive killing effect in the combination group, not the least killing of mutant strains. However, this is a key point for us, and is included in future planned studies.

## Conclusion

In conclusion, our study provides evidence that locally applied immunomodulatory S100A8/A9 as an adjunct to established antibiotics could hinder resistance development and reduce the pathogenic bacterial burden in infected wounds. Our present results and previous observations on S100A8/A9 indicate a clinical role for S100A8/A9 in handling medical biofilm infected chronic wounds.

## Data Availability Statement

The original contributions presented in the study are included in the article/supplementary material. Further inquiries can be directed to the corresponding author.

## Ethics Statement

The animal study was reviewed and approved by Animal Ethics Committee of Denmark, approval number 2015-15-0201-00618.

## Author Contributions

All authors contributed to conceptualization, visualization, data curation validation, methodology and writing, review and editing. CM and NH were main supervisors. All authors contributed to the article and approved the submitted version.

## Funding

CM is the receiver of the Borregaard Clinical Ascending Investigator grant from Novo Nordisk [NNF17OC0025074]. The funder was not involved in the study design, collection, analysis, interpretation of data, the writing of this article or the decision to submit it for publication. AL has received a fully funded Ph.D. scholarship by the research committee at Rigshospitalet [E-22416-05]. The data has not been presented at any previous meetings.

## Conflict of Interest

The authors declare that the research was conducted in the absence of any commercial or financial relationships that could be construed as a potential conflict of interest.
